# NUAK1 acts as a novel regulator of PD-L1 via activating GSK-3β/β-catenin pathway in hepatocellular carcinoma

**DOI:** 10.1186/s10020-025-01088-7

**Published:** 2025-02-03

**Authors:** Chao-Yan Yao, Hang-Tian Tao, Jin-Jin He, Feng-Yi Zhu, Cui-Qing Xie, Yu-Na Cheng, Ji-Qin Li, Zhuang-Zhuang Liu, Chun-Yu Hou, Xue-Li Liu, Yong-Li Fan, Dong Fang, Xin-Rui Lv

**Affiliations:** 1 Department of Pharmacy, The First Affiliated Hospital of Henan University, Kaifeng, 475004 China; 2 Institute of Chemical Biology, School of Pharmacy, Henan University, N. Jinming Ave, Kaifeng, 475004 China; 3 Department of Oncology, The First Affiliated Hospital of Henan University, Kaifeng, 475000 China; 4 Kaifeng Key Laboratory for Infectious Diseases and Biosafety, The First Affiliated Hospital of Henan University, Ximen Ave, Kaifeng, 475000 China

**Keywords:** NUAK1, GSK3β, Β-catenin, PD-L1, Hepatocellular carcinoma

## Abstract

**Background:**

NUAK1 is associated with metastasis and drug resistance in hepatocellular carcinoma (HCC). However, little is known about the immune functions of NUAK1 in HCC. Therefore, the aim of this study was to elucidate the novel role of NUAK1 in facilitating immune evasion in HCC and to investigate the mechanisms underpinning this process.

**Method:**

The levels of NUAK1 expression and the infiltration of CD8^+^ T cells were assessed in tumor tissues from HCC patients and mice xenograft model. HCC cell lines were used to validate the role of NUAK1 in regulating the transcription of PD-L1, the diethylnitrosamine-induced HCC model was established and the expression levels of NUAK1 and PD-L1 proteins in the rat livers were detected. Western blotting, immunofluorescence, real time PCR, and immunohistochemical staining were used to investigate the underlying mechanisms by which NUAK1 regulates PD-L1 expression in hepatocellular carcinoma.

**Results:**

NUAK1 expression was negatively correlated with CD8^+^ T cell infiltration in tumor tissues from HCC patients and mice xenograft model. Both gain and loss of functions have identified NUAK1 promoted PD-L1 expression at transcriptional level in HCC cells. The increased expression of NUAK1 and PD-L1 proteins were observed in the rat livers of diethylnitrosamine-induced HCC model. Moreover, overexpression of NUAK1 promotes GSK3β Ser^9^ phosphorylation, β-catenin expression and nuclear accumulation in HCC cells. By contrast, knockdown of NUAK1 has opposite effects. Inhibition of GSK3β activity significantly promoted β-catenin expression and PD-L1 expression in HCC cells. IHC analyses of tumor tissues from HCC patients suggested that the levels of p-GSK3β and β-catenin were positively correlated with NUAK1 expression. Knockdown of β-catenin also reversed NUAK1-mediated PD-L1 expression in HCC cells.

**Conclusions:**

This study revealed a novel role for NUAK1, which promotes the transcriptional expression of PD-L1 by activating GSK3β/β-catenin signaling pathway, leading to immune escape of hepatocellular carcinoma.

*Registry and the registration no. of the study/trial*: Not applicable.

**Supplementary Information:**

The online version contains supplementary material available at 10.1186/s10020-025-01088-7.

## Introduction

Programmed death ligand-1 (PD-L1, encoded by CD274 gene) is highly expressed in a variety of human cancers (Zou et al. 2023; Yamaguchi et al. 2022). As an important immune checkpoint protein, PD-L1 on the tumor cell surface directly interacts with programmed death 1 (PD-1) on the T cell, which can suppress T-cell activity and proliferation, thereby resulting in immune escape during tumorigenesis (Yu et al. 2020). Recently, immune checkpoint blockade (ICB) targeting PD-L1/PD-1 blocking therapy has achieved some success in many malignancies including non-small-cell lung cancer (NSCLC) and melanoma (Punekar et al. 2022; Jacquelot et al. 2017).

In patients with unresectable hepatocellular carcinoma, atezolizumab combined with bevacizumab therapy showed better overall and progression-free survival outcomes than sorafenib (Finn et al. 2020). Unfortunately, only 16–20% of hepatocellular carcinoma (HCC) patients respond to PD1/PD-L1 blocking therapy (Fu et al. 2019; Cheng et al. 2020). Studies have shown that the expression level of PD-L1 on HCC cells is a determinant of the therapeutic effect of PD-L1/PD-1 (Wu et al. 2019). Therefore, understanding the regulatory mechanism of PD-L1 in HCC is of great significance for the identification of new immune predictors and improve the response rate of PD1/PD-L1 blocking therapy.

The novel (nua) kinase family 1 (NUAK1), which has been originally identified as the fifth member of the AMP-activated protein kinase (AMPK) catalytic subunit family, is a serine/threonine kinase (Humbert et al. 2010). Elevated NUAK1 expression has been observed in hepatocellular carcinoma, gastric cancer, ovarian cancer, glioma and other tumors (Faisal et al. 2021; Yao et al. 2023; Lu et al. 2013). Tumor patients with high expression of NUAK1 often have poor prognosis and reduced overall survival (Lu et al. 2013; Phippen et al. 2016). NUAK1 was reported to promote tumor growth and metastasis through inducing cytoskeleton rearrangement and matrix metalloproteinase (MMP) activation in cancer cells (Suzuki et al. 2004). Knockdown of NUAK1 increased the antitumor efficacy of doxorubicin by inhibiting epithelial-mesenchymal transition in HCC (Xu et al. 2016). Moreover, depletion or inhibition of NUAK1 prolongs animal survival in mouse models of different tumors, indicating NUAK1 is a potential therapeutic target (Liu et al. 2012).

Despite accumulating evidence revealed the direct role of NUAK1 on cancer cell proliferation, migration and drug resistance, it is still unknown whether NUAK1 contributes to immune escape in HCC. In the present study, we aimed to investigate the role of NUAK1 in tumor immune microenvironment and explore its potential mechanism in HCC.

## Materials and methods

### Reagents and antibodies

Diethylnitrosamine (DEN), SB216763, and lithium chloride were purchased from Sigma-Aldrich (Saint Louis, MO, USA). Rabbit monoclonal anti**-**PD-L1 antibody (ab228415, 1:1000 for western blot), rabbit anti-PD-L1 antibody (ab233482, 1:200 for immunohistochemistry), rabbit anti-β-catenin (ab32572, 1:500 for immunofluorescence or immunohistochemistry), rabbit anti-CD8α (ab217344, 1:200 for immunofluorescence or immunohistochemistry), rabbit anti-GZMB (ab255598, 1:500 for immunofluorescence) were purchased from Abcam (Cambridge, MA, USA). Rabbit anti-NUAK1 antibody (4458s, 1:1000 for Western blot), rabbit anti-p-GSK3β antibody (Ser^9^) (5558T, 1:500 for Western blot; 1:200 for immunohistochemistry), rabbit anti-Lamin B1 (13435 S, 1:500 for western blot) were purchased from Cell Signaling Technology (Beverly, MA, USA). Rabbit anti-GSK3β antibody (sc-9166, 1:1000 for western blot) and mouse anti-β-actin antibody (sc-47778, 1:2000 for western blot) were purchased from Santa Cruz Biotechnology (Santa Cruz, CA, USA). Rabbit anti-β-catenin antibody (66379-1-Ig, 1:1000 for western blot) and rabbit anti-NUAK1 antibody (22723-1-AP, 1:200 for immunofluorescence or immunohistochemistry) were purchased from Protein Tech Group (Chicago, IL, USA). Horseradish peroxidase (HRP)-conjugated goat anti-rabbit immunoglobulin G (IgG) (ZB-2301, 1:1000 for western blot) and goat anti-mouse IgG (ZB-2305, 1:1000 for western blot) were purchased from Zhongshan Golden Bridge Company (Beijing, China). APC/Cyanine7 anti-mouse CD45 antibody (103116, 1:50 for flow cytometry), PE/Cyanine7 anti-mouse CD8α (100722, 1:50 for flow cytometry), APC anti-mouse CD3 antibody (100236, 1:50 for flow cytometry), FITC anti-mouse GZMB ((372206, 1:50 for flow cytometry), TruStain FcX™ PLUS (anti-mouse CD16/32) antibody (156603, 1:200 for flow cytometry) were purchased from BioLegend (San Diego, CA, USA).

### Cell lines and culture

All cell lines used in the experiments were purchased from the Cell Bank of the Chinese Academy of Science (Shanghai, China) and were periodically authenticated by short tandem repeat with a multi-amplification kit (PowerPlex 16HS System). They were routinely cultured in DMEM supplemented with 10% fetal bovine serum at 37 °C in a humidified incubator containing 5% CO_2_.

### Plasmids construction and transfection

PCR-amplified full-length β-catenin cDNA was inserted into the pCMV-Flag-His-puro vector, and the targeted shRNA sequences used to knock down of β-catenin was cloned into the pLKO.1-puro vector. For transient transfection, 2 µg plasmids were transfected into HCC cells using Lipofectamine 2000 DNA Transfection Reagent (Invitrogen, Carlsbad, CA, USA). After 4 h of transfection, the medium was replaced with fresh medium for further culture for 24 h. Cells were collected for further experiments. The target sequences of human β-catenin shRNA were as follows: shRNA-1: 5′-GCC ATG GAA CCA GAC AGA AA-3′, shRNA-2: 5′-AAG TCC TGT ATG AGT GGG AAC-3′.

To establish stable transduction, lentiviruses containing full-length NUAK1 sequence or short hairpin RNA of NUAK1 were designed and produced by Genechem company (Shanghai, China). NUAK1 cDNA was inserted into Ubi-MCS-CBh-gcGFP-IRES-puromycin vector, and the targeted shRNA sequences used to knock down of NUAK1 were cloned into the hU6-MCS-CBh-gcGFP-IRES-puromycin vector. HCC cell lines were infected with Lentiviruses in the presence of 8 µg/mL polybrene (Millipore, Burlington, MA, USA). After 72 h, the cells were selected in the presence of 1.5 µg/mL puromycin (Sigma, St. Louis, USA) for 1 week, and finally, the cells were collected for further experiments. The target sequences of NUAK1 shRNA were as follows: human NUAK1-shRNA-1: 5′-TCG ATG ACA ACT GCA ATAT-3′, human NUAK1-shRNA-2: 5′-CAT CCT CAT ATC ATC AGTA-3′, human NUAK1-NC: 5′-TTC TCC GAA CGT GTC ACGT-3′. Mouse NUAK1-shRNA-1: 5′-GAT GTG ACT CAG GTG TATA-3′, mouse NUAK1-shRNA-2: 5′-AGC TAG ACA TGG TTC ACAT-3′, mouse NUAK1-NC: 5′-TTC TCC GAA CGT GTC ACGT-3′.

### Bioinformatic analysis

The expression levels of NUAK1 mRNA in normal tissues (Normal) and HCC (Tumor) were examined utilizing data from the Gene Expression Omnibus (GEO) database (https://ncbi.nlm.nih.gov/geo). Specifically, the dataset GSE14520 was accessed from GEO. The analysis of differential NUAK1 expression was conducted using R software (version 3.5.0) and the Wilcoxon test, with statistical significance defined as a P-value less than 0.05. Additionally, a Kaplan-Meier survival analysis was performed to assess overall survival (OS) in HCC patients, stratified by high and low NUAK1 expression, using the median expression level as the cutoff. Statistical analyses were conducted utilizing the double-tailed log-rank test. The relationship between NUAK1 mRNA expression levels and the abundance of CD8^+^ T cells, as well as CD274 mRNA expression in HCC, was examined using data from The Cancer Genome Atlas (TCGA) database (https://tcga-data.nci.nih.gov/tcga/) and analyzed with R software version 3.5.0. The correlation was assessed using Spearman’s rho coefficient, with a P-value of less than 0.05 indicating statistical significance. The Wilcoxon test, implemented in R software version 3.5.0, was employed to analyze PD-L1 mRNA expression in HCC patients from the TCGA database, stratified by high and low NUAK1 expression levels. The effect of NUAK1 expression on CD274 mRNA expression was also evaluated, with a P-value of less than 0.05 deemed statistically significant.

### Western blot analysis

The total and nuclear proteins were extracted as previously described (Yang et al. 2023). Protein samples were denatured and separated by SDS-polyacrylamide gel electrophoresis. The PVDF membranes were then incubated with primary antibodies at 4 °C overnight and HRP-conjugated secondary antibodies for 1 h at room temperature, The blots were detected using the enhanced chemiluminescence plus reagents and visualized using a ECL Plus™ reagents.

### Reverse transcription-quantitative (RT-q) PCR

Total RNA extraction, reverse transcription, and real-time PCR were performed as described previously (Shi et al. 2022). Glyceraldehyde-3-phosphate dehydrogenase (GAPDH) was used as an internal control. Specific primers for human HCC cells were as follows: CD274: 5′-TGG CAT TTG CTG AAC GCA TTT-3′(forward) and 5′-TGC AGC CAG GTC TAA TTG TTTT-3′ (reverse); GAPDH: 5′-GAC ACC CAC TCC TCC ACC TTT-3′(forward) and 5′-TTG CTG TAG CCA AAT TCG TTGT-3′(reverse). Specific primers for rat were as follows: NUAK1:5′-ATG CCC TTC GAT GGC TTT GA-3′(forward) and 5′-CGA GCA TCT GAG GGT TGT GT-3′(reverse), CD274: 5′-TTA TAG TCA CAG CCT GCA GTC AGG − 3′5′-TTA TAG TCA CAG CCT GCA GTC AGG − 3′(forward) and 5′-ATC GTG ACA TTG CTG CCA TAC TC −3′ (reverse); GAPDH, 5′-AGC CAT GTA CGT AGC CAT CC-3′ (forward) and 5′-GCC ATC TCT TGC TCG AAG TC-3′(reverse).

### Animal experiments

Male Sprague-Dawley rats aged 6 weeks and male BALB/c mice aged 5 weeks were provided by Beijing Weitong Lihua Animal Co. All animal protocols were approved by the Institutional Animal Care and Use Committee of Henan University (HUSOM2021-196). The animals were housed under an environmentally controlled conditions (temperature 23 ± 2 °C, relative humidity 60 ± 5%, and a 12 h dark/light cycle).

To induce HCC, the rats were intraperitoneally injected with diethylnitrosamine (DEN) at a dose of 50 mg/kg once a week for 16 weeks. Rats in the control group were intraperitoneally injected with normal saline solution on the same days as the DEN-treated group.

For the in vivo tumorigenesis experiment, BALB/c mice were divided into five groups: H22/vector, H22/NUAK1-OE (NUAK1 overexpression), H22/sh-vector, and H22/sh-NUAK1-1^#^. H22/sh-NUAK1-2^#^. A total of 2 × 10^6^ cells were subcutaneously injected into the left dorsal flank of each mouse. Eight mice per group in each experiment were included. The tumors were allowed to grow for 2 weeks, and then, the mice were euthanized by cervical dislocation. Tumor tissues were excised for immunohistochemistry (IHC) staining and flow cytometric analysis.

### Human tissue specimens

A total of 20 HCC tissues were collected from the first affiliated hospital of Henan University between 2020 and 2021. The present study was approved by both the Institute Research Ethics Committee of the first affiliated hospital of Henan University, and informed consent was obtained from all enrolled patients.

### Immunohistochemistry (IHC) staining

Human HCC tissues and xenograft tumor tissues were fixed with 10% formalin and embedded with paraffin. The paraffin-embedded tissues were cut into 4 μm sections and placed on polylysine-coated slides. Immunohistochemistry was performed as described previously (Niu et al. 2020).

### Immunofluorescence (IF)

For immunofluorescence staining of cultured HCC cells, Adherent cells were fixed in 4% paraformaldehyde at room temperature for 15 min, permeabilized in 0.1% Triton X-100 for 10 min at 4 ℃. Cells were stained with rabbit anti-β-catenin overnight at 4 °C, followed by incubation with Alexa Fluor 555-conjugated secondary antibodies for 1 h at room temperature. Nuclei were stained with 4,6-diamidino v-2-phenylindole (DAPI, Life Technologies). Images were acquired using an LSM 510 M confocal microscope (Carl Zeiss).

For multiple immunofluorescence, the TSA Plus fluorescence three-standard four-color staining kit (Servicebio) was utilized to sequentially detect NUAK1, CD8α, and GZMB. Initially, paraffin-embedded sections of mouse tumors underwent dewaxing with xylene followed by gradient rehydration using ethanol. Subsequently, the sections were immersed in a 3% hydrogen peroxide solution to inactivate endogenous peroxidase activity and subjected to antigen retrieval. This was followed by a blocking treatment, after which the sections were incubated sequentially with rabbit anti-NUAK1 antibody (1:200), rabbit anti-CD8α antibody (1:200), rabbit anti-GZMB antibody (1:200), and rabbit HRP. After each incubation period with the secondary antibody, the staining solutions TSA-488, TSA-555, and TSA-647 were applied for fluorescence labeling. Each primary antibody was labeled individually. Post-staining, antigen retrieval was performed once using a sodium citrate buffer in a microwave oven, followed by washing with PBS. After three cycles of antibody incubation and fluorescent labeling, the nuclei were counterstained with DAPI. Images were acquired using a laser confocal microscope after the samples were mounted, the quantification of mean fluorescence intensity of NUAK1, CD8, and GZMB in individual cells was conducted using ImageJ software.

### Flow cytometric analysis

For the analysis of tumor-infiltrating lymphocytes, tumors were minced with scissors and digested with 1 mg/mL of collagenase digestion solution for 1 h in a shaker at 37 °C. Single cell suspension was obtained by rapid and gentle stripping. After blocking with CD16/CD32 (40477) antibody, the cells were stained with APC-Cy7-CD45, APC**-**CD3, PE-CY7**-**CD8α, for 30 min at 4 °C in the dark. After fixation and permeabilization by True-Nuclear Transcription factor Buffer Set, intracellular GZMB was stained using FITC-GZMB antibody. After washed three times with PBS, the stained cells were suspended in PBS and examined using a BD FACS Aria II flow cytometer (BD Bioscience, San Jose, CA, USA). Data were further analyzed by Flow Jo 10.0 software.

### Statistical analysis

All analyses were performed using GraphPad Prism 5 for Windows (GraphPad 10 Software, Inc., La Jolla, CA, USA). All data were expressed as the mean ± SEM. The statistical significance of the difference between the two groups was determined using an unpaired two-tailed Student’s t-test, whereas one-way analysis of variance (ANOVA) followed Tukey post hoc tests was used to compare the mean values of multiple experimental groups. Pearson’s correlation test was used to examine the correlation of two proteins, *p* < 0.05 was considered to indicate a statistically significant difference.

## Results

### NUAK1 promotes tumor immune escape by inhibiting CD8+ T cell infiltration in HCC

**Fig. 1:**
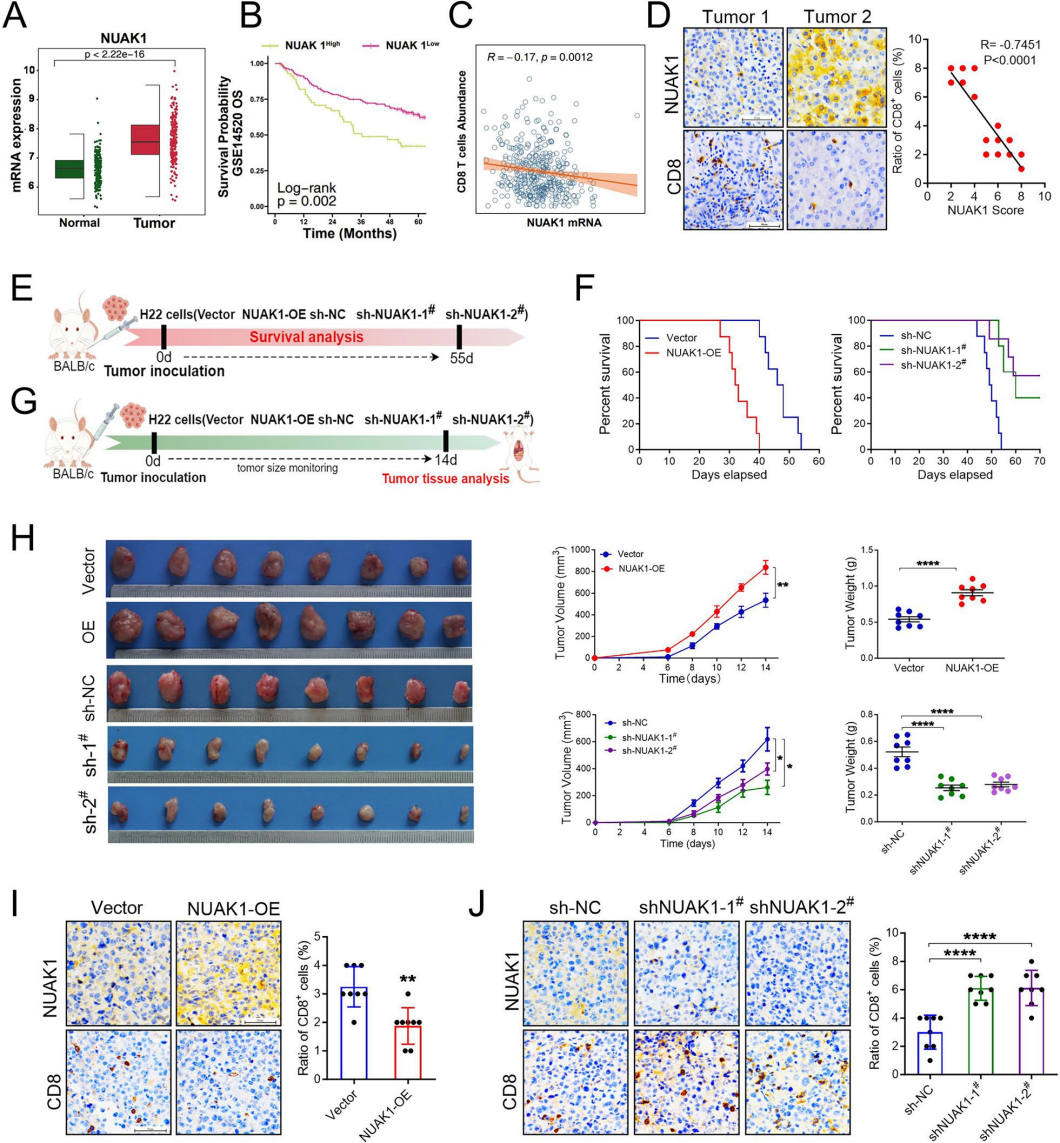
NUAK1 promotes tumor immune escape by inhibiting CD8^+^ T cell infiltration in HCC. **A** The expression of NUAK1 in normal (*n* = 212) and tumor (*n* = 222) tissues in HCC was evaluated based on the GEO database. The X-axis represents normal and HCC (Tumor), and the Y-axis represents the expression level of NUAK1 mRNA. **B** Kaplan–Meier overall survival curves of HCC (*n* = 370, cutoff = 50%) patients with of high and low NUAK1 mRNA expression obtained from the GSE14520. The X-axis represents survival time (months), and the Y-axis represents the patient’s probability of survival. **C** Correlation analysis of NUAK1 mRNA level and the abundance of CD8^+^ T cells in HCC (*n* = 370) based on the RNA-seq datasets derived from the TCGA database. The X axis represents the expression level of NUAK1 mRNA, and the Y axis represents the abundance of CD8^+^T cells. **D** Immunohistochemical staining of NUAK1 and CD8^+^ in 20 human HCC specimens. Left: Representative images of two tumors. Scale bar, 50 μm. Right: Pearman correlation test between tumoral NUAK1 and CD8^+^ IHC scores. Note that the scores of some samples overlap. **E** Schematic diagram of experimental protocol for survival. **F** Kaplan-Meier survival curves of vector and NUAK1-overexpression (NUAK1-OE) and sh-vector (sh-NC) and two sh-NUAK1 (1^#^ and 2^#^)-transfected H22 xenografts in BALB/c mice, *n* = 8 mice per group. **G** Schematic diagram of experimental protocol for tumor analysis. **H** Representative images of tumors, quantification of tumor volume and tumor weight collected from vector, NUAK1-OE and sh-NC, two sh-NUAK1 groups, *n* = 8. **p < 0.01, ***p < 0.001, ****p < 0.0001, one-way ANOVA. **I** IHC staining of tumoral NUAK1 and CD8^+^ from tumor-grafted mice constructed vector or NUAK1-OE H22 cells, *n* = 8. **p < 0.01, two tailed unpaired t test per group. **J** IHC staining of tumoral NUAK1 and CD8^+^ from tumor-grafted mice constructed sh-NC and two sh-NUAK1 (1^#^ and 2^#^) H22 cells, *n* = 8. ***p < 0.001, one-way ANOVA

To assess the role of NUAK1 in immune escape in hepatocellular carcinoma, we initiated our study by analyzing the mRNA expression of NUAK1 in HCC and its prognostic significance using the Gene Expression Omnibus (GEO) database. The outcomes indicated that the expression of NUAK1 was dramatically elevated in tumor tissues, and the over-all survival (OS) of patients with high NUAK1 expression was significantly lower than that of patients with low NUAK1 expression (Fig. 1A, B). we performed correlation analyses on the expression profile of NUAK1 mRNA and the abundance of CD8^+^T cells in 370 HCC samples based on The Cancer Genome Atlas (TCGA) database, and the results demonstrated that the expression level of NUAK1 mRNA was inversely correlated with the abundance of CD8^+^T cells in HCC samples (Fig. 1C). In order to further verify this result, we examined the expression level of NUAK1 and CD8α in HCC patients. Through immunohistochemical (IHC) staining, we found that the tumor tissues with a higher expression of NUAK1 had a decreased CD8^**+**^ T cell infiltration, Pearson correlation analysis showed NUAK1 expression was negatively correlated with CD8^**+**^ T cell infiltration (Fig. 1D). Next, we constructed a subcutaneous xenograft tumor model using NUAK1-overexpression and NUAK1-silencing H22 cells to evaluate the role of NUAK1 in HCC. Kaplan-Meier Plotter assay showed that NUAK1 overexpression significantly shortened the OS of tumor-bearing mice, whereas NUAK1 knockdown exhibited opposite effects (Fig. 1E, F). By measuring the tumor volume and weight, we found that NUAK1 overexpression dramatically promoted tumor growth in vivo, while knockdown of NUAK1 showed opposite effects on tumor growth (Fig. 1G, H).

**Fig. 2:**
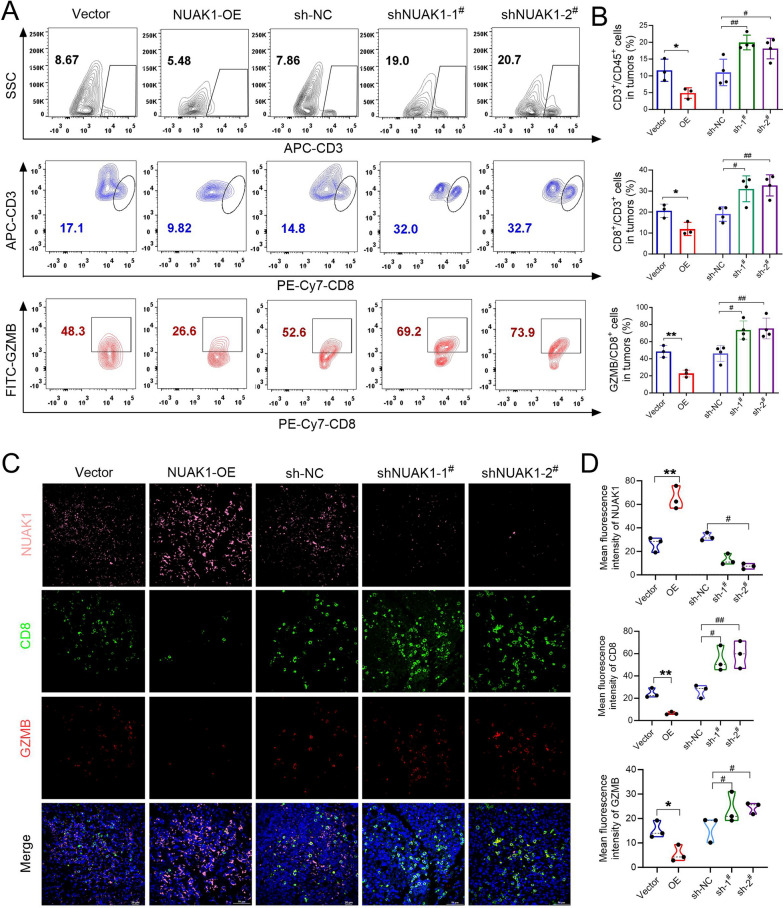
NUAK1 inhibits CD8^+^ T cell infiltration and activity in HCC. **A**,** B** Representative flow cytometry plots showing CD3^+^, CD8^+^ and CD8^+^GZMB^+^ T cells from Vector, NUAK1-OE, Sh-NC, sh-NUAK1-1^#^, sh-NUAK1-2^#^ tumors (**A**) and quantification (**B**). *p < 0.05, **p < 0.01 compared with vector group, two tailed unpaired t test per group, *n* = 3; ^#^*p* < 0.05, ^##^*p* < 0.01, ^###^*p* < 0.001 compared with sh-NC group; one-way ANOVA, *n* = 4. **C**,** D** Immunofluorescence staining and quantitative statistics of NUAK1 (pink), CD8α (green) and GZMB (red) of tumoral NUAK1 and CD8^+^ from tumor-grafted mice constructed Vector, NUAK1-OE, sh-NC, sh-NUAK1-1^#^, sh-NUAK1-2^#^ H22 cells. Scale bars, 50 μm. *p < 0.05, **p < 0.01 compared with vector group, two tailed unpaired t test per group; ^#^*p* < 0.05, ^##^*p* < 0.01 compared with sh-NC group; one-way ANOVA, *n* = 3

Subsequently, we conducted IHC staining to examine the effects of NUAK1 on intratumoral CD8^+^ T cell infiltration in tumor-bearing mice. As expected, overexpression of NUAK1 significantly reduced infiltration of CD8^+^ T cells in xenograft tumors (Fig. 1I). However, knockdown of NUAK1 significantly enhanced the infiltration of CD8^+^ T cells in xenograft tumors (Fig. 1J). Consistent with these findings, flow cytometry analysis further confirmed that overexpression of NUAK1 significantly reduced both the population and activity (GZMB^+^) of CD8^+^ T cells within the tumor. Conversely, the depletion of NUAK1 notably increased both the quantity and activity (GZMB^+^) of CD8^+^ T cells in the tumor (Fig. 2A, B). Additionally, the results from the multiple immunofluorescence analysis of NUAK1, CD8α, and GZMB demonstrated that the overexpression of NUAK1 markedly decreased the population of GZMB^+^ CD8^+^ T cells within the tumor microenvironment. In contrast, the deletion of NUAK1 resulted in a significant increase in the population of GZMB^+^ CD8^+^ T cells in the tumor region. (Fig. 2C, D). Collectively, these findings indicated that NUAK1 inhibits CD8^+^ T cell infiltration and activity in tumor microenvironment.

### NUAK1 induces tumor immune escape by promoting PD-L1 expression

**Fig. 3:**
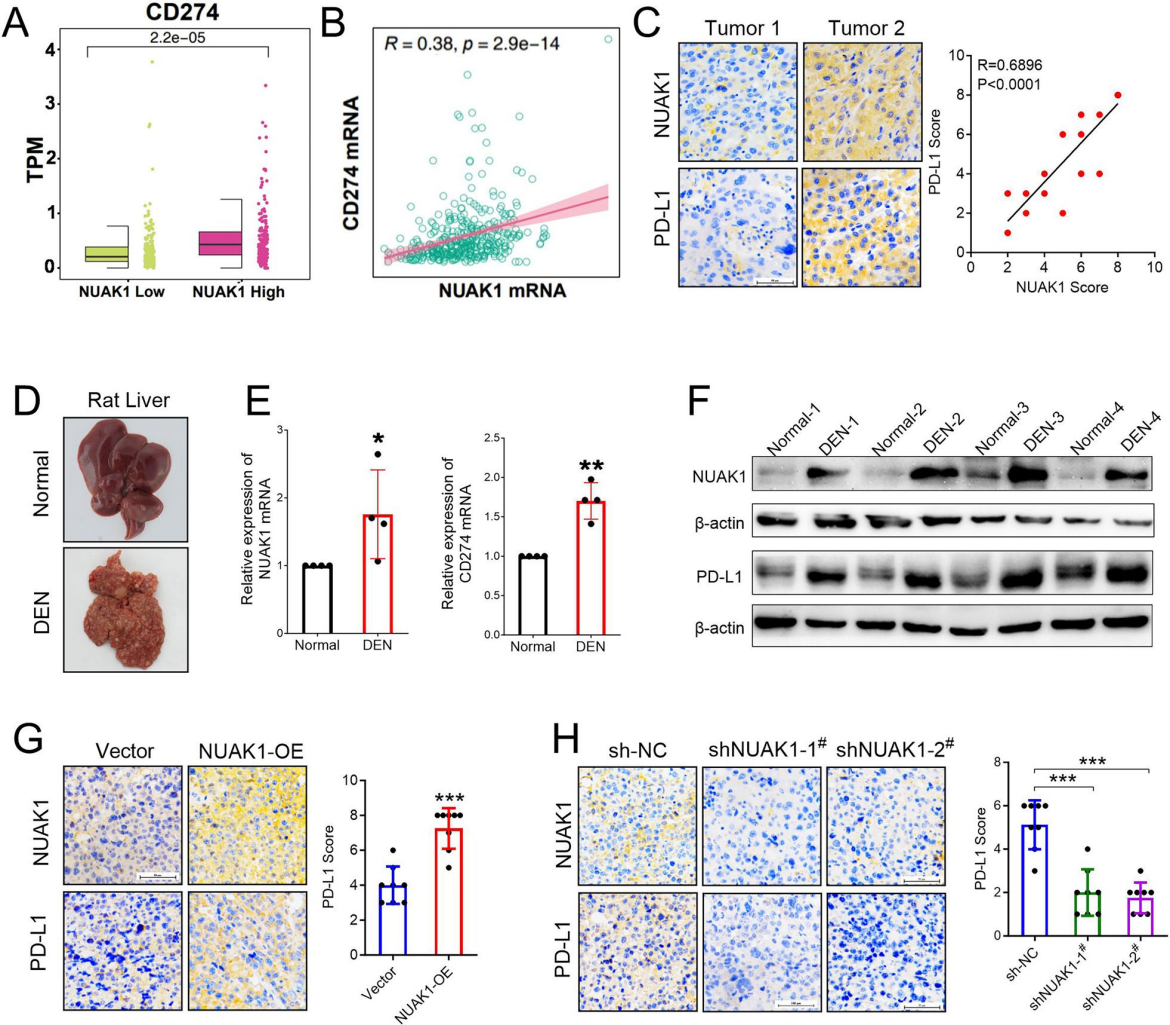
NUAK1-mediated tumor immune escape is associated with PD-L1 expression **A** The expression levels of CD274 mRNA were evaluated in HCC with high (*n* = 364) and low (*n* = 364) NUAK1 mRNA expression from the TCGA database. The X axis represents the group with high or low expression of NUAK1, and the Y axis represents the expression level of CD274 mRNA using TPM (Transcripts Per Kilobase of exon model per Million mapped reads). **B** Correlation analysis of NUAK1 mRNA and of PD-L1 mRNA level in HCC (*n* = 370) based on the RNA-seq datasets derived from the TCGA database. The X axis represents the expression level of NUAK1 mRNA, and the Y-axis represents the expression level of CD274 mRNA. **C** Immunohistochemical staining of NUAK1 and PD-L1 in 20 human HCC specimens. Left: Representative images of two tumors. Right: Pearman correlation test between tumoral NUAK1 and PD-L1 IHC scores, note that the scores of some samples overlap. **D** Representative images of livers from normal and DEN-treated rats. **E**,** F** The mRNA (**E**) and protein (**F**) expression of NUAK1 and PD-L1 in the livers of normal rats and DEN-tread rats, *n* = 4. **p < 0.01, ***p < 0.001, two tailed unpaired t test. **G** IHC staining of tumoral NUAK1 and PD-L1 from tumor-grafted mice constructed with or without NUAK1-OE H22 cells, *n* = 8. ***p < 0.001, two tailed unpaired t test. **H** IHC staining of tumoral NUAK1 and PD-L1 from tumor-grafted mice constructed with or without NUAK1-knockdown H22 cells, *n* = 8. ***p < 0.001, one-way ANOVA

To explore whether NUAK1 mediates the immune escape of hepatocellular carcinoma through inducing PD-L1, we conducted an analysis of the expression of CD274 in HCC samples featuring low and high expression of NUAK1, and the correlation between the expression of NUAK1 and CD274 mRNA level was detected using the TCGA database. The results indicated that the expression of NUAK1 and PD-L1 was positively correlated (Fig. 3A, B). Furthermore, we detected the expression of NUAK1 and PD-L1 in HCC patients by IHC staining, and found that tumor tissues with high NUAK1 expression also had high PD-L1 expression. Pearson correlation analysis showed that NUAK1 expression was positively correlated with PD-L1 expression (Fig. 3C). To further demonstrate these results, we established a rat model of hepatocellular carcinoma by intraperitoneal injection of diethylnitrosamine (DEN) (Fig. 3D). Then, the expression levels of NUAK1 and PD-L1 in rat liver were detected. As the results shown, compared with the normal group, the mRNA and protein expression of NUAK1 and PD-L1 in the rat livers of DEN group were significantly increased (Fig. 3E, F). Similarly, in subcutaneous xenograft tumor model, IHC staining suggested that overexpression of NUAK1 greatly promoted PD-L1 expression (Fig. 3G). Conversely, knockdown of NUAK1 significantly inhibited the expression of PD-L1 (Fig. 3H).

**Fig. 4:**
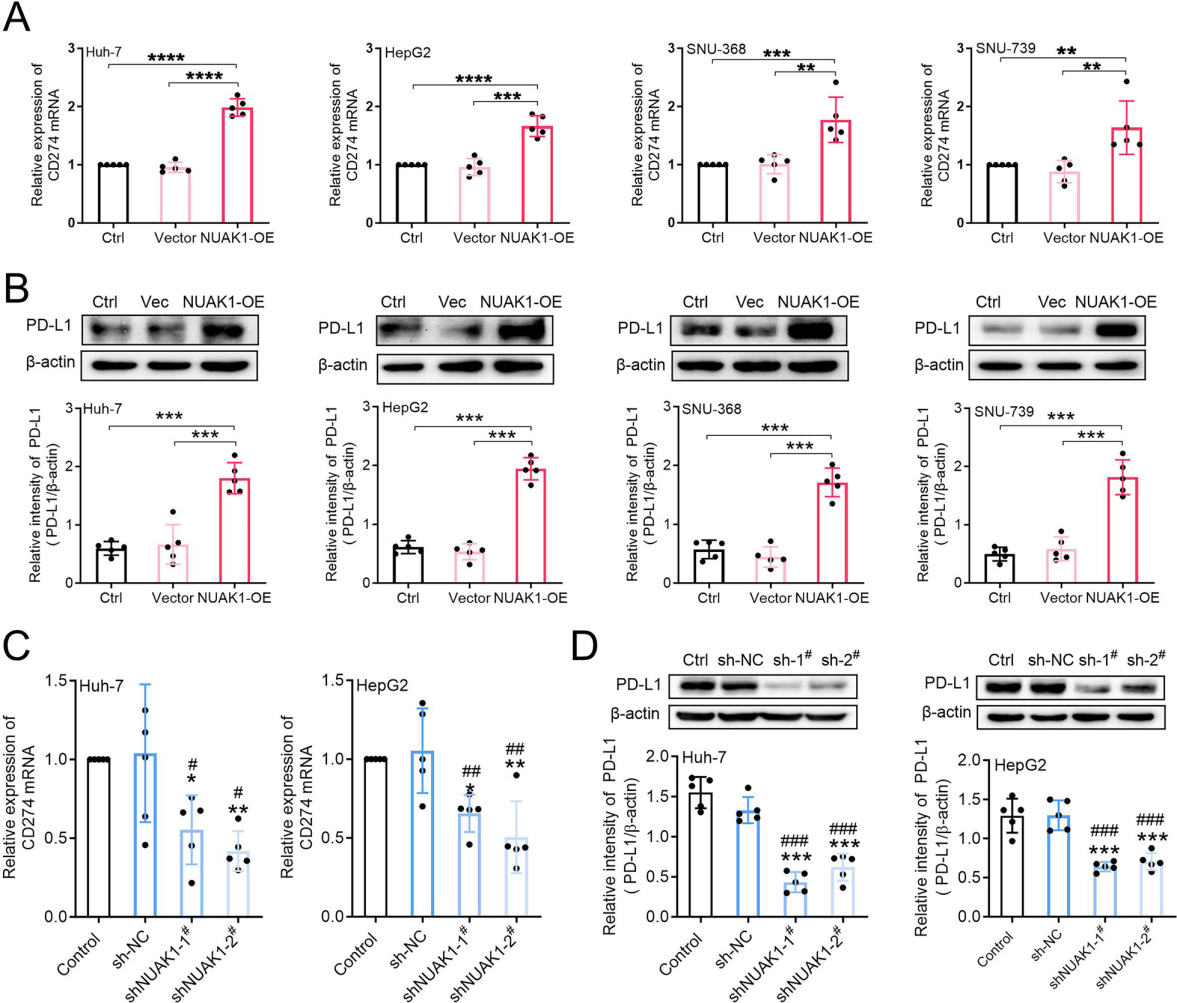
NUAK1 promotes PD-L1 expression in HCC cells. **A**,** B** The effects of NUAK1 overexpression on the levels of CD274 mRNA (A) and PD-L1 protein (B) in HCC cells, *n* = 5. **p < 0.01, ***p < 0.001, one-way ANOVA. **C**,** D** Effects of NUAK1 knockdown on CD274 mRNA (C) and PD-L1 protein (D) expression in Huh-7 and HepG2 cells, *n* = 5. *p < 0.05, **p < 0.01, ***p < 0.001 compared with control group; ^#^*p* < 0.05, ^##^*p* < 0.01, ^###^*p* < 0.001 compared with sh-NC group; one-way ANOVA

Next, we constructed stable NUAK1-overexpressing HCC cells to investigate whether NUAK1 could promote PD-L1 expression. The results showed that ectopic expression of NUAK1 significantly promoted CD274 mRNA and PD-L1 protein expression in Huh-7, HepG2, SNU-368 and SNU-739 cells (Fig. 4A, B). In contrast, knockdown of NUAK1 with two specific shRNAs dramatically reduced CD274 mRNA and PD-L1 protein expression in Huh-7 and HepG2 cells (Fig. 4C, D). Collectively, these results indicated that NUAK1 can up-regulate the expression level of PD-L1 at the transcriptional level, and ultimately promote immune escape of HCC.

### NUAK1 promotes PD-L1 expression by inactivating glycogen synthase kinase 3β in HCC

**Fig. 5:**
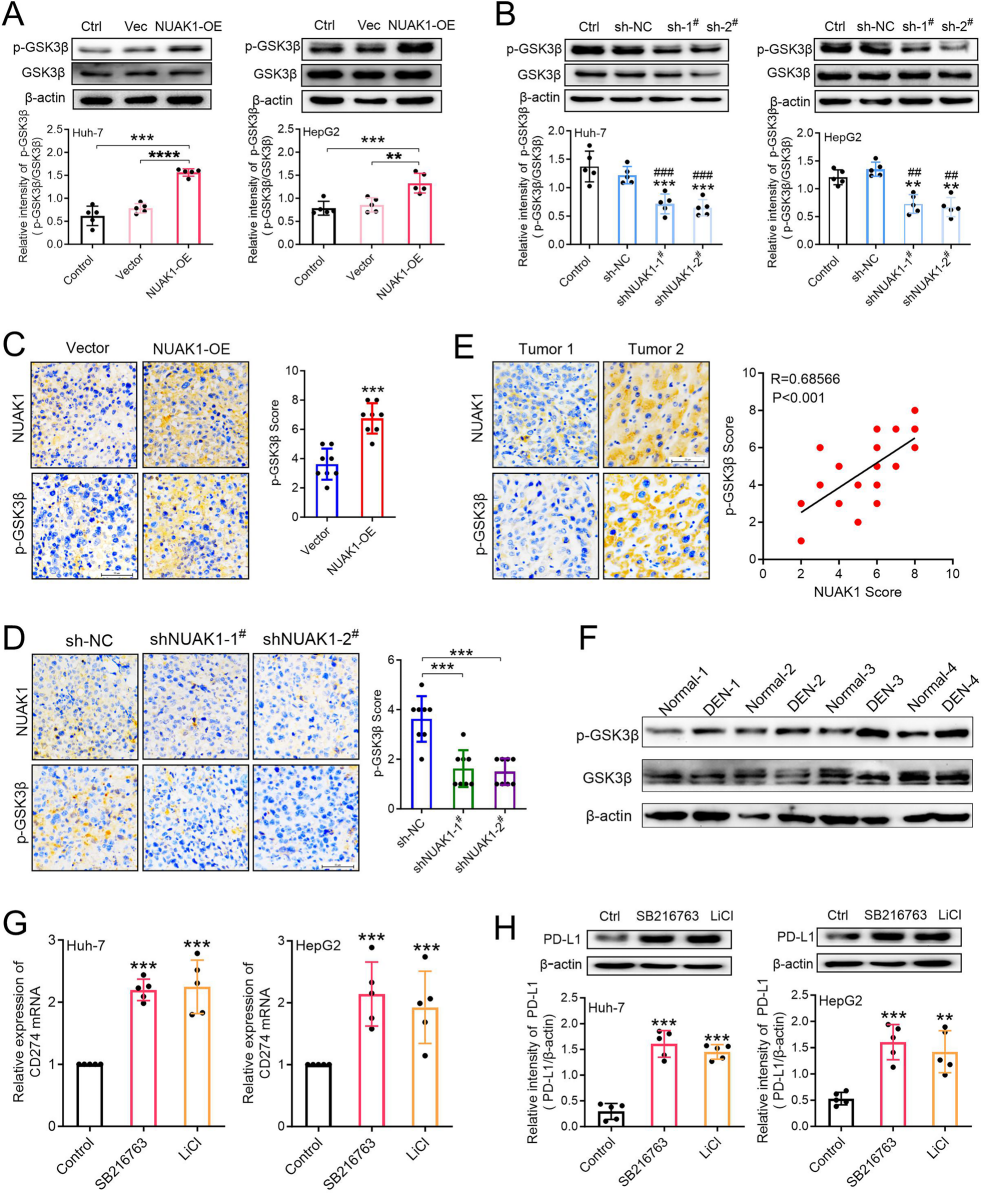
NUAK1 induces phosphorylation of GSK3β at the Ser9 residue, and inhibition of GSK3β promotes PD-L1 expression in HCC. **A** Effects of NUAK1 overexpression on the phosphorylation level of GSK3β at Ser^9^ in Huh-7 and HepG2 cells, *n* = 5. **p < 0.01, ***p < 0.001, one-way ANOVA. **B** Effects of NUAK1 knockdown on the phosphorylation level of GSK3β at Ser^9^ in Huh-7 and HepG2 cells, *n* = 5. **p < 0.01, ***p < 0.001 compared with control group; ^##^*p* < 0.01, ^###^*p* < 0.001 compared with sh-NC group; one-way ANOVA. **C** IHC staining of tumoral NUAK1 and p-GSK3β from tumor-grafted mice constructed with or without NUAK1-overexpressing H22 cells. Left: Representative images. Scale bar, 50 μm. Right: quantification analysis. *n* = 8, ***p < 0.001, two tailed unpaired t test. **D** IHC staining of tumoral NUAK1 and p-GSK3β from tumor-grafted mice constructed with or without NUAK1-knockdown H22 cells. *n* = 8, ***p < 0.001, one-way ANOVA. **E** Immunohistochemical staining of NUAK1 and p-GSK3β in 20 human HCC specimens. Left: Representative photos of two tumors. Right: Pearman correlation test between tumoral NUAK1 and p-GSK3β. Note that the scores of some samples overlap. **F** The protein expression of p-GSK3β in the livers of normal rats and DEN-tread rats (*n* = 4). **G**,** H** Effects of GSK3β inhibitors SB216763 and lithium chloride (LiCl) on CD274 mRNA (**G**) and PD-L1 protein (**H**) expression in Huh-7 and HepG2 cells. *n* = 5, ***p < 0.001, one way ANOVA

NUAK1 has been reported to inactivate glycogen synthase kinase-3 beta (GSK3β) by suppressing the de-phosphorylation pathway of GSK3β Ser^9^ in colorectal cancer cells (Yang et al. 2021). Therefore, we hypothesize that NUAK1 promotes the transcriptional expression of PD-L1 by inactivating GSK3β in HCC cells. To prove this hypothesis, we first measured the phosphorylation level of GSK3β at Ser^9^, an indicator of GSK3β inactivation, in both NUAK1 overexpressed and knockdown cells. As expected, ectopic expression of NUAK1 dramatically promotes phosphorylation of GSK3β Ser^9^ in Huh-7 and HepG2 cells (Fig. 5A), whereas knockdown of NUAK1 significantly decreased the phosphorylation of GSK3β Ser^9^ in Huh-7 and HepG2 cells (Fig. 5B). In support of our in vitro results, IHC analyses of subcutaneous xenograft tumors suggested that overexpression of NUAK1 greatly promoted p-GSK3β expression (Fig. 5C). Conversely, knockdown of NUAK1 significantly inhibited the expression of p-GSK3β (Fig. 5D). IHC analyses of tumor tissues from 20 HCC patients suggested that the levels of p-GSK3β were positively correlated with NUAK1 expression (Fig. 5E). What’s more, we also observed an increased expression of p-GSK3β in the livers of DEN treated rats (Fig. 5F).

Subsequently, we used two specific GSK3β inhibitors, 10 µM SB216763 or 2 mM lithium chloride to treat Huh-7 and HepG2 cells for 24 h and examined PD-L1 expression. The results showed that both SB216763 and lithium chloride dramatically promoted CD274 mRNA and PD-L1 protein expression in Huh-7 and HepG2 cells (Fig. 5G, H). Together, these data suggested that NUAK1 upregulates PD-L1 by inactivating GSK3β in HCC.

### NUAK1 promotes PD-L1 expression in a GSK3β/β-catenin dependent manner in HCC

**Fig. 6:**
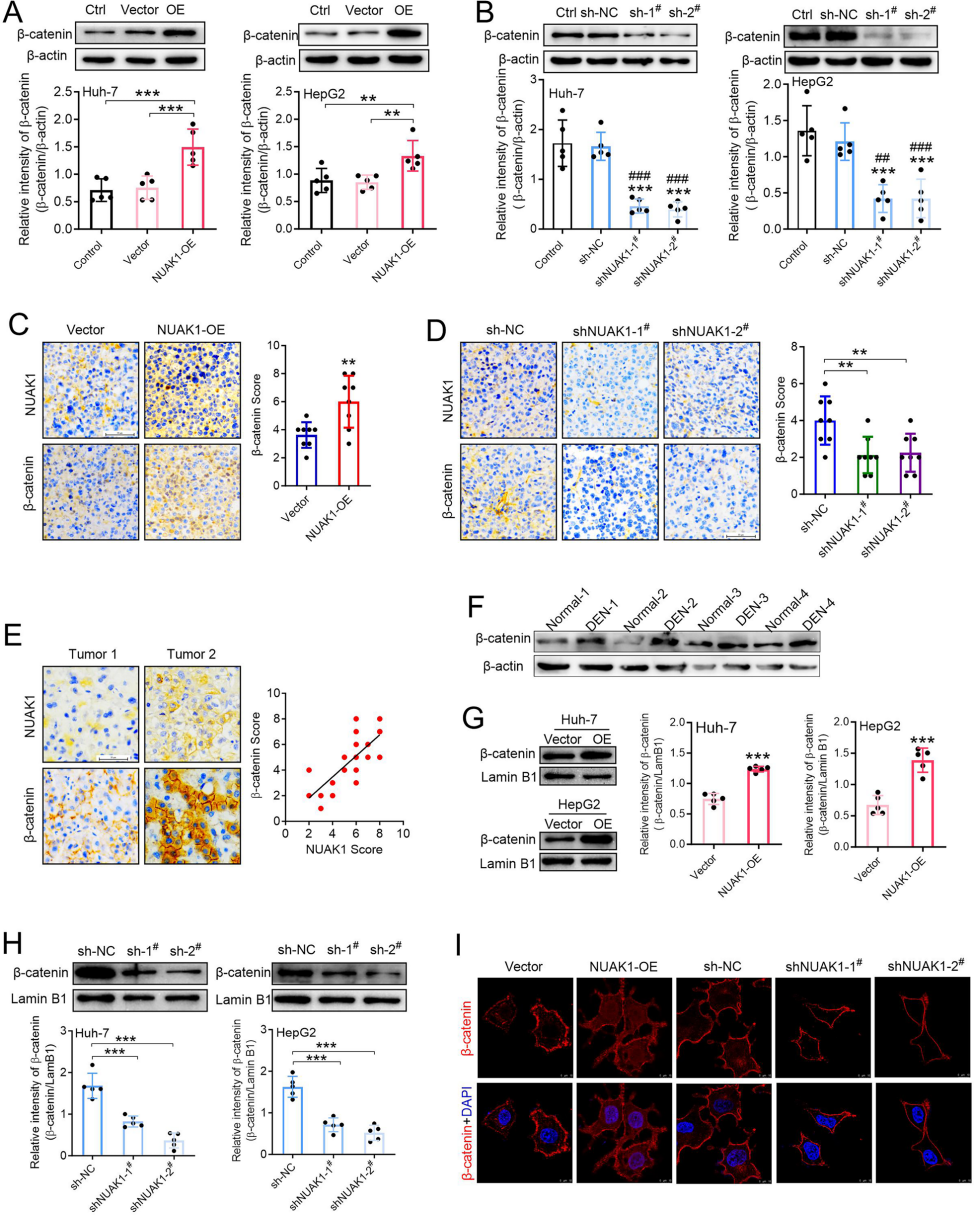
NUAK1 promotes β-catenin expression and nuclear accumulation in HCC **A** Effects of NUAK1 overexpression on the expression of β-catenin total protein in HCC cells. *n* = 5, **p < 0.01, ***p < 0.001, one-way ANOVA. **B** Effects of NUAK1 knockdown on the β-catenin total protein expression in HCC cells. *n* = 5, ***p < 0.001 compared with Ctrl group; ^##^*p* < 0.01, ^###^*p* < 0.001 compared with sh-NC group; one-way ANOVA. **C** IHC staining of tumoral NUAK1 and β-catenin from tumor-grafted mice constructed with or without NUAK1-overexpressing H22 cells. *n* = 8, *p < 0.05, two tailed unpaired t test. **D** IHC staining of tumoral NUAK1 and β-catenin from tumor-grafted mice constructed with or without NUAK1-knockdown H22 cells. Data were expressed by mean ± SEM (*n* = 8). *p < 0.05, two-tailed unpaired t test. **E** Pearman correlation test between tumoral NUAK1 and p-GSK3β in 20 human HCC specimens. Note that the scores of some samples overlap. **F** The protein expression of β-catenin in the livers of normal rats and DEN-tread rats (*n* = 4). **G** Effects of NUAK1 overexpression on the β-catenin nuclear protein expression in Huh-7 and HepG2 cells. Lamin B1 was used as internal control. *n* = 5, ***p < 0.001, two-tailed unpaired t test. **H** Effects of NUAK1 knockdown on the β-catenin nuclear protein expression in Huh-7 and HepG2 cells. Lamin B1 was used as internal control. Data were expressed by mean ± SEM (*n* = 5). ***p < 0.001; one-way ANOVA. **I** Immunofluorescence staining of β-catenin in Huh-7 cells stably transfected with vector, NUAK1-overexpression (NUAK1-OE), sh-Vector (sh-NC), NUAK1-knockdown^#^1 (sh-NUAK1^#^1) and NUAK1-knockdown^#^2 (sh-NUAK1^#^2) groups

As one of the main components of β-catenin-destruction complex, GSK-3β can phosphorylate β-catenin which results in its proteasomal degradation (Kimelman and Xu 2006). Given that NUAK1 can inhibit the activity of GSK3β by promoting the phosphorylation of GSK3β at serine^9^, we first examined the effects of NUAK1 on β-catenin expression in HCC cells. The results indicated that NUAK1 overexpression greatly increased the total β-catenin expression (Fig. 6A). In contrast, knockdown of NUAK1 inhibited its total protein expression in Huh-7 and HepG2 cells (Fig. 6B). By analyzing xenograft tumors, we found that NUAK1 overexpression greatly promoted β-catenin expression (Fig. 6C), knockdown of NUAK1 significantly inhibited β-catenin expression (Fig. 6D). IHC analyses of tumor tissues from 20 HCC patients suggested that the levels of β-catenin were positively correlated with NUAK1 expression (Fig. 6E). Furthermore, Western blotting detected an increased expression of β-catenin in the livers of DEN-treated rats (Fig. 6F).

β-Catenin in the cytoplasm usually translocates into the nucleus where it is activates acts as a transcriptional co-activator (Kypta and Waxman 2012). Therefore, we examined the effects of NUAK1 on nuclear β-catenin expression. As expected, ectopic expression of NUAK1 greatly increased the nuclear expression of β-catenin in Huh-7 and Hep G2 cells (Fig. 6G). while knockdown of NUAK1 inhibited β-catenin expression in the nucleus (Fig. 6H). By immunofluorescence, we further confirmed these results (Fig. 6I).

**Fig. 7:**
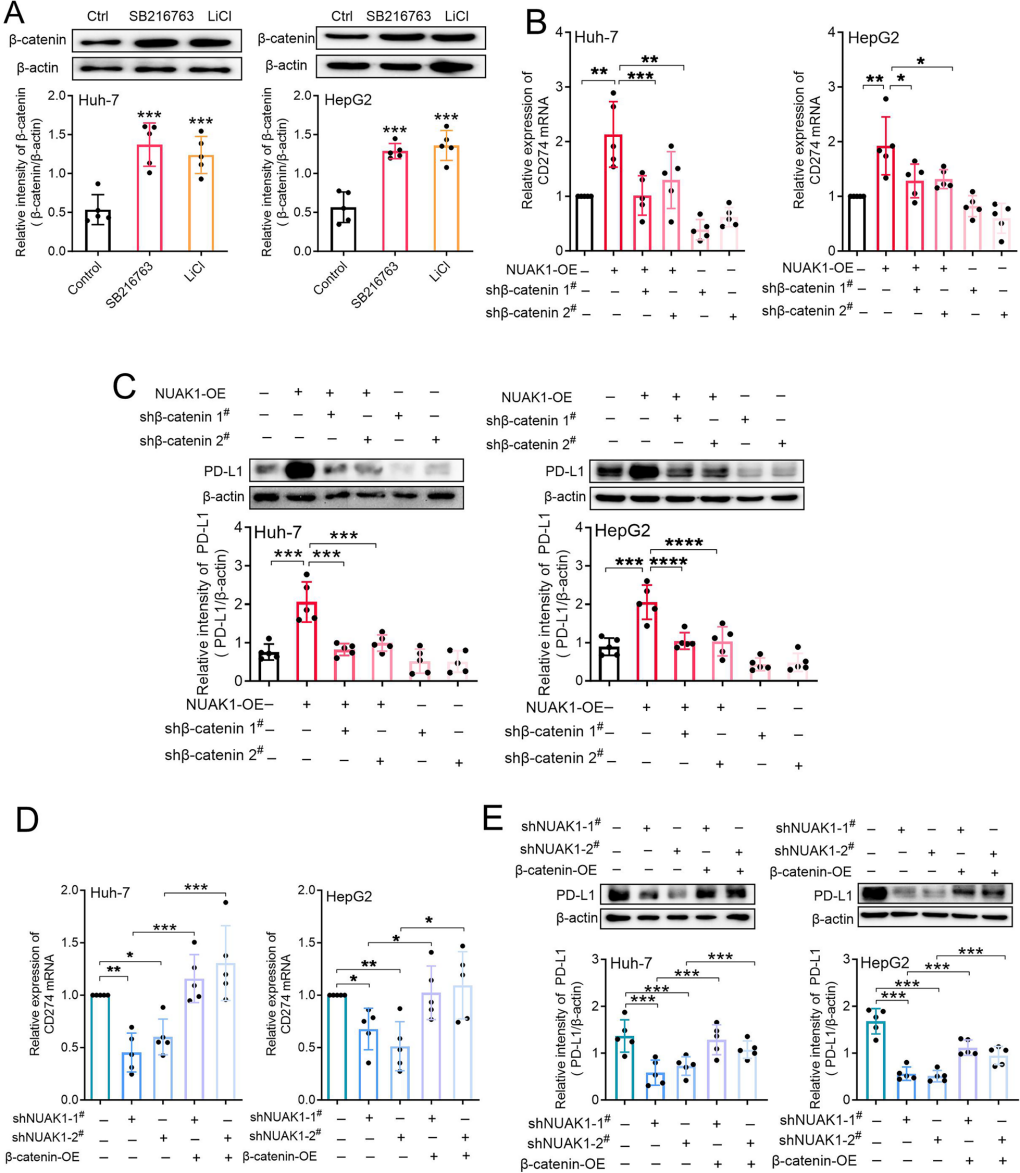
NUAK1 promotes PD-L1 expression in a GSK3β/β-catenin dependent manner in HCC. **A** Effects of GSK3β inhibitors SB216763 and LiCl on β-catenin total protein expression in HCC cells. *n* = 5, ***p < 0.001, one-way ANOVA. **B**,** C** Effects of β-catenin knockdown on NUAK1 overexpression-induced CD274 mRNA (**B**) and PD-L1 protein (**C**) expression in HCC cells. *n* = 5, *p < 0.05, **p < 0.01, ***p < 0.001, one-way ANOVA. **D**,** E** Effects of β-catenin overexpression on NUAK1 knockdown-induced CD274 mRNA (**D**) and PD-L1 protein (**E**) expression in Huh-7 and HepG2 cells. *n* = 5, *p < 0.05, **p < 0.01, ***p < 0.001, one-way ANOVA

Finally, to verify that NUAK1 promotes the PD-L1 expression through GSK3β/β-catenin pathway, we first treated Huh-7 and HepG2 cells with 10 µM SB216763 or 2 mM lithium chloride for 24 h and examined β-catenin expression. As expected, these two specific GSK3β inhibitors significantly promoted β-catenin expression in HCC cells (Fig. 7A). Then, we knocked down of β-catenin in NUAK1-overexpressed Huh-7 and HepG2 cells and examined PD-L1 expression. As expected, knockdown of β-catenin using two specific shRNA significantly blocked NUAK1 overexpression-induced CD274 mRNA and PD-L1 protein expression in Huh-7 and HepG2 cells (Fig. 7B, C). Subsequently, we overexpressed β-catenin and examined its effect on PD-L1 expression in NUAK1-knockdown hepatocellular carcinoma cell lines. RT-qPCR and Western blot analysis suggested that overexpression of β-catenin significantly reversed NUAK1 knockdown-induced the down-regulation of CD274 mRNA and PD-L1 protein in HCC cells (Fig. 7D, E).

## Discussion

Overexpression of NUAK1 has been detected in a variety of tumors and is associated with tumor progression (Lu et al. 2013; Phippen et al. 2016). Here, our study first provided novel insights into the role of NUAK1 on tumor immune evasion. As one of AMPK-related protein kinase (ARK) family members, NUAK1 has been reported to inhibit protein phosphatase 1β (PP1β) activity by phosphorylating PP1β regulatory subunit myosin phosphatase targeting-1 (MYPT1) (Zagorska et al. 2010; Angulo-Urarte et al. 2018). Given that PP1β could dephosphorylate GSK3β at the inhibitory Ser^9^ site (Port et al. 2018), we therefore supposed that NUAK1 promoted GSK3β phosphorylation at Ser^9^ site through inhibiting PP1β activity in HCC. In support of our assumption, Port et al. found that NUAK1 in colorectal cancer cells suppressed PP1β-dependent de-phosphorylation of GSK3β in response to oxidative stress (Port et al. 2018). The inactivation of GSK3β by Ser^9^ residue phosphorylation usually loses its ability to phosphorylate β-catenin, leading to nuclear accumulation and transcriptional activity of β-catenin (Vangamudi et al. 2011). The GSK-3β/β-catenin pathway has been reported to be involved in a variety of important cellular functions and is abnormally activated in a variety of malignancies, leading to tumor progression (Pan et al. 2021; Song et al. 2020). Consistent with these reports, we observed that inhibition of the phosphorylation of GSK3β Ser^9^ significantly reduced β-catenin expression in HCC cells. It is noteworthy that GSK3β phosphorylates the Thr^180^ and Ser^184^ residues of non-glycosylated PD-L1 and induces PD-L1 proteasome degradation through the β-transducin repeat-containing protein (β-TRCP) pathway (Li et al. 2016). Although our study demonstrated that GSK3β regulates the expression of PD-L1 at the transcriptional level and revealed another form of regulation of GSK3β on PD-L1 expression, we cannot rule out the possibility that NUAK1 could inhibit PD-L1 proteasome degradation through inhibiting the activity of GSK3β in HCC cells.

The expression level of PD-L1 is a determining factor affecting the efficacy of PD1/PD-L1 immunotherapy, and the regulatory mechanisms of PD-L1 at the transcriptional and post-translational modification levels have been extensively studied (Yamaguchi et al. 2022; Zhou et al. 2022). At the transcriptional level, several transcription factors including signal transducer and activator of transcription 3 (STAT3), c-myc, bromodomain containing 4 (BRD4), nuclear factor-kB (NF-κB) can regulate the expression of PD-L1 by binding to DNA-regulatory sequences (Atsaves et al. 2017; Zhu et al. 2016). After translation, newly-synthesized PD-L1 protein requires a series of post-translational modifications (PTMs) to perform its biological function. The PTMs of PD-L1 refers to glycosylation, ubiquitination, phosphorylation, acetylation, and palmitoylation, which is involved in the occurrence and development of tumors (Hsu et al. 2018). In this study, our findings revealed that NUAK1 can promote transcriptional expression of PD-L1 by activating the GSK3β/β-catenin signaling pathway, providing a novel strategy to improve the efficacy of PD-1/PD-L1 immunotherapy in HCC patients.

The upregulation of PD-L1 is regarded as a mechanism facilitating tumor immune escape through the inhibition of CD8^+^ T cell activity. This mechanism has been extensively demonstrated in multiple tumor types. In this study, we discovered that NUAK1 induces the expression of PD-L1 at the transcriptional level, thereby reducing the infiltration and activity of CD8^+^ T cells in tumor tissues. It is notable that Tan et al. found that NUAK1 was positively correlated with the number of CD8^+^ T cells in the study of idiopathic pulmonary fibrosis (IPF), but the precise mechanism of action remains to be further explored (Tan et al. 2024). This disparity between tumor and fibrotic diseases might be associated with the distinct disease types and the diversity of T cell differentiation. In fibrotic diseases such as IPF, CD8^+^ T cells infiltrate the lung tissue and differentiate into phenotypes with opposing effects on fibrosis: IFN-γ-producing cells that may ameliorate fibrosis and IL-4-producing cells that may exacerbate it (Walker et al. 1994). This indicates that CD8^+^ T cells assume a dual role in fibrosis, contingent upon the differentiation phenotype, which might either inhibit or facilitate the fibrotic process. However, the specific differentiation phenotype of CD8^+^ T cells induced by NUAK1 in the tumor microenvironment awaits investigation in our future work. We believe that a deeper comprehension of this mechanism will assist in revealing the complex regulatory role of NUAK1 on CD8^+^ T cell function in diverse pathological settings, and subsequently provide a theoretical foundation for the development of novel therapeutic strategies.

β-Catenin is highly expressed in the tumor tissues of HCC patients and plays a vital role in tumor progression (Xu et al. 2022; Morita et al. 2023). On the one hand, β-catenin promotes cancer cell proliferation and migration by inducing c-myc, cyclin D1, and MMPs expression (Li et al. 2005). On the other hand, activation of β-catenin pathway was determined to induce transcriptional expression of PD-L1 in HCC cells, resulting in reduction in CD8^+^ T cell infiltration in the tumor microenvironment (Yang et al. 2023; Shi et al. 2022). In the present study, we found that knockdown of β-catenin significantly blocked NUAK1-induced PD-L1 at transcriptional level in HCC cells, highlighting the significance of β-catenin in tumor immune escape. Although the direct roles of NUAK1 on cancer cell proliferation and migration were determined in HCC (Yao et al. 2023), the underlying mechanisms remains largely unknown. Based on our results, we suggested that NUAK1 may promote HCC cell proliferation and migration by inducing β-catenin nuclear translocation in vitro. Notably, β-catenin can promote transcriptional expression of N-glycosyltransferase STT3, which in turn enhances PD-L1 glycosylation and stabilization in HCC cells (Shi et al. 2022). Therefore, it raises the possibility that NUAK1 promote PD-L1 glycosylation by inducing β-catenin/STT3 signaling pathway in HCC cells.

**Fig. 8:**
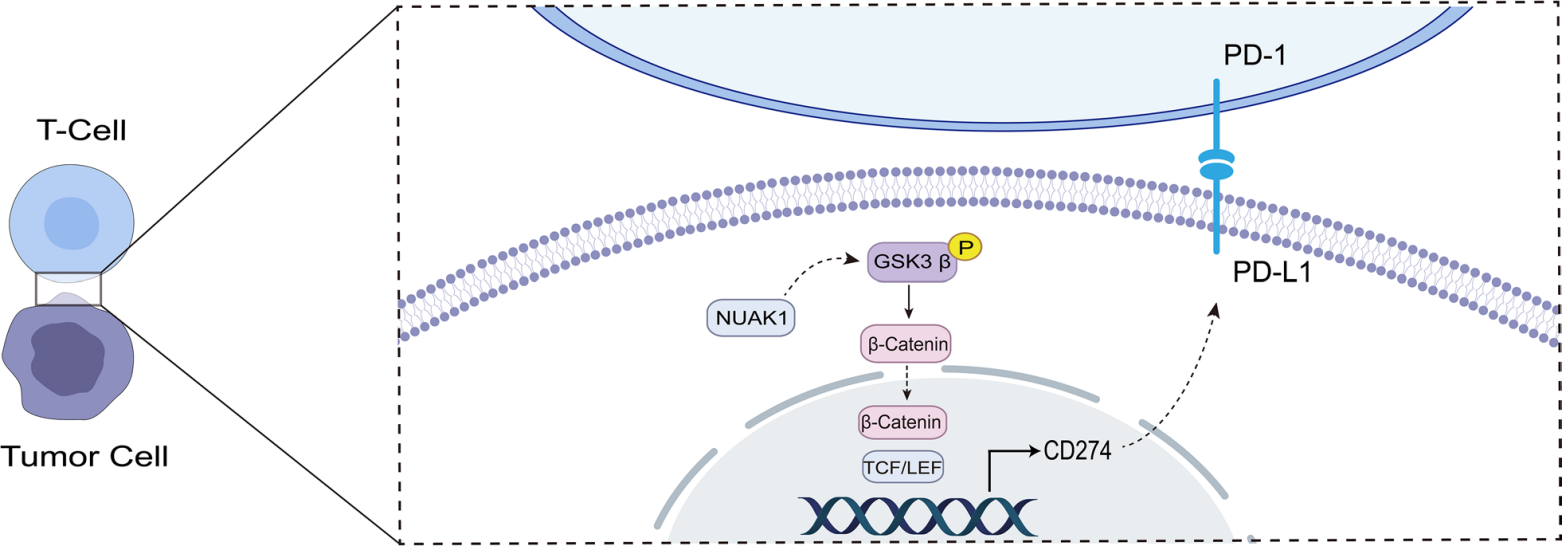
Schematic representation of NUAK1-mediated PD-L1 expression in HCC. NUAK1 enhances the transcriptional expression of PD-L1 by activating the GSK3β/β-catenin signaling pathway, thereby suppressing cytotoxic T cell activity within the tumor microenvironment and facilitating immune evasion in hepatocellular carcinoma

In conclusion, NUAK1 promotes PD-L1 expression through the activation of GSK3β/β-catenin pathway, thereby reducing the infilitration and activity of CD8+ T cells and leading to immune escape of HCC (Fig. 8). Our results reveal a new role of NUAK1 in tumor immune regulation, which has important implications for the identification of new immune predictors and the development of more predictors and therapeutic markers for HCC.

## Supplementary Information

Supplementary Material 1.

## Data Availability

No datasets were generated or analysed during the current study.
