# Supplementary material for: NUAK1 acts as a novel regulator of PD-L1 via activating GSK-3β/β-catenin pathway in hepatocellular carcinoma
Source: Mol Med. 2025 Feb 3;31:38. doi: 10.1186/s10020-025-01088-7 (PMC11789290; doi:10.1186/s10020-025-01088-7)
Supplement: Supplementary file 1 — Supplementary Material 1. [file 10020_2025_1088_MOESM1_ESM.docx]

**
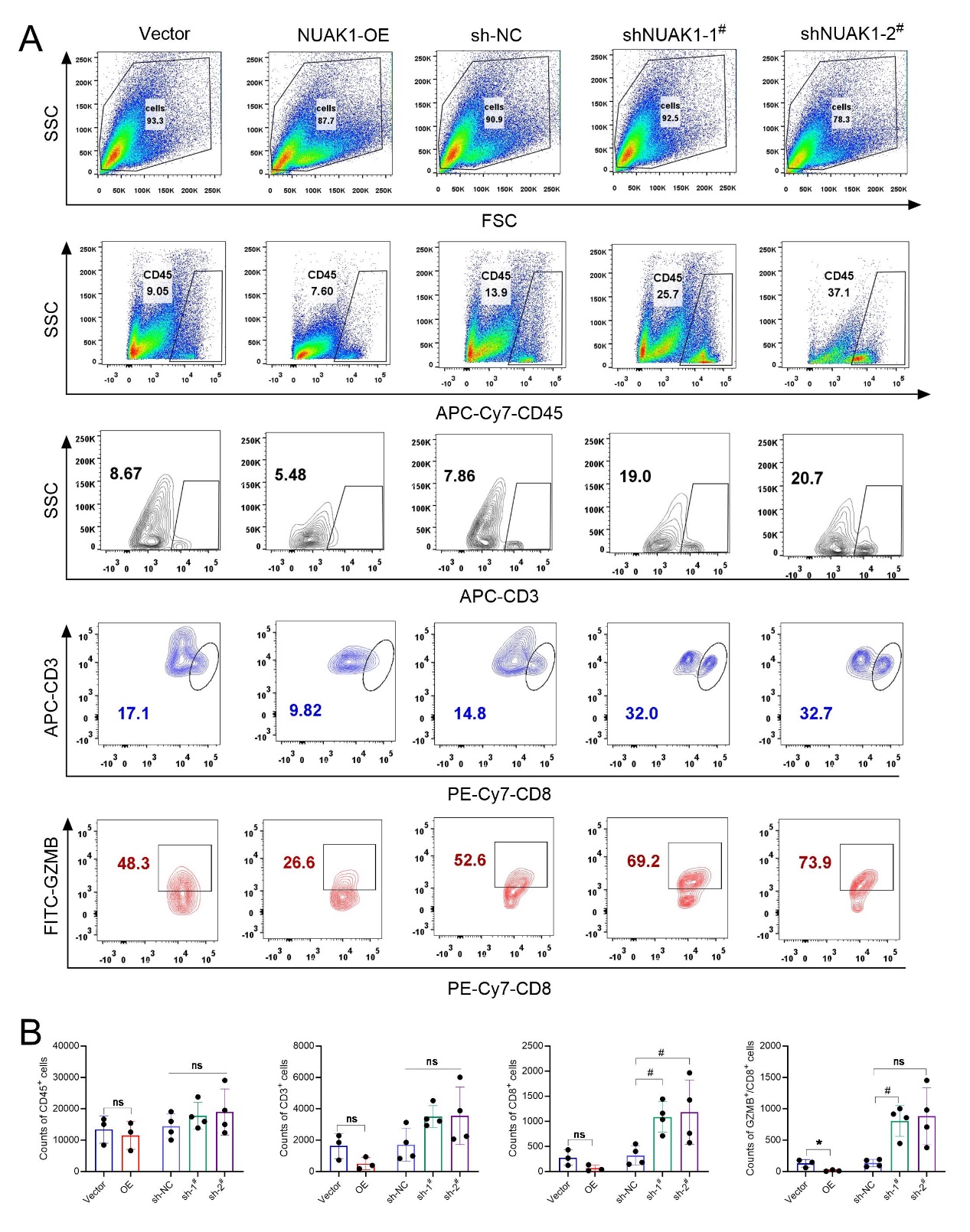
**

**Fig S1, related to Fig 2**

(A) The gating strategy for identifying CD3^+^ cells within the CD45^+^ cell population, CD8^+^ cells within the CD3^+^ cell population and GZMB^+^ cells within the CD8^+^ T cell subset was examined across Vector, NUAK1-OE, Sh-NC, sh-NUAK1-1^#^, and sh-NUAK1-2^#^ tumor samples. (B) The enumeration of CD45^+^ CD3^+^, CD8^+^ and GZMB^+^/CD8^+^ cells within a population of 10^5^ tumor cells. *p < 0.05 compared with vector group, two tailed unpaired t test per group, n=3; #p < 0.05 compared with sh-NC group; one-way ANOVA, n=4.

**Western blot images**

**Figure 3**


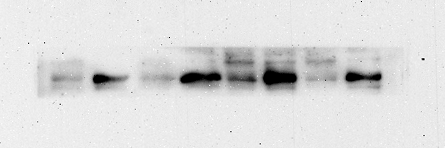
**F**

NUAK1


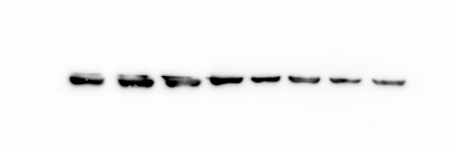


β-actin


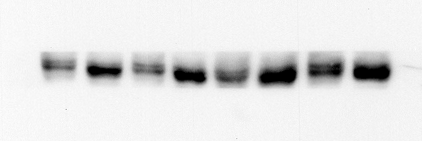


PD-L1


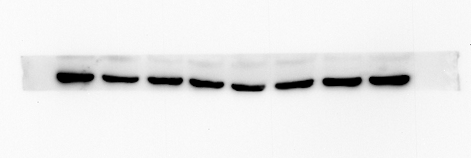


β-actin

**Figure 4**


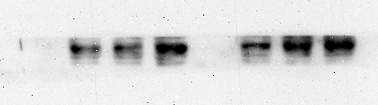
**B**

PD-L1

**(Huh-7)**


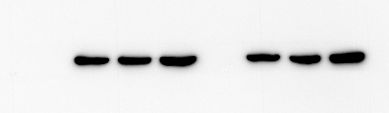


β-actin


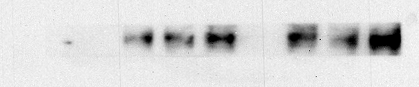


PD-L1

**(HepG2)**


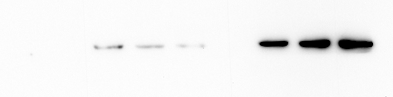


β-actin


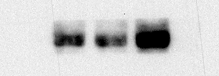
PD-L1

**(SNU-368)**


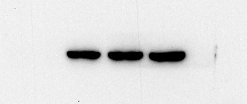


β-actin


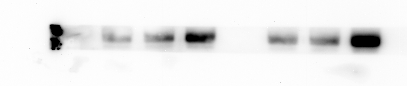


PD-L1


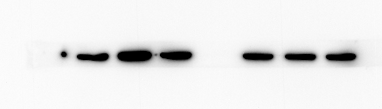


**(SNU-739)**

β-actin


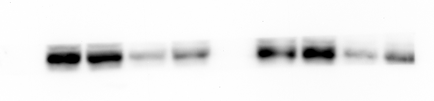
**D**

PD-L1

**(Huh-7)**


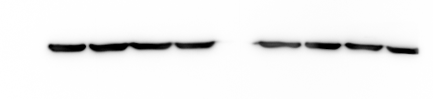


β-actin


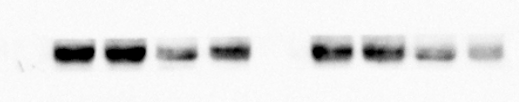


PD-L1

**(HepG2)**


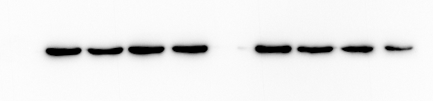


β-actin

**Figure 5**


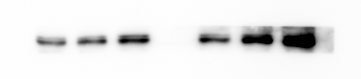
**A**

p-GSK3β


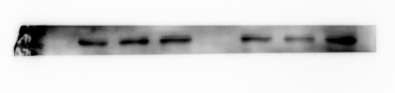


**(Huh-7)**

GSK3β


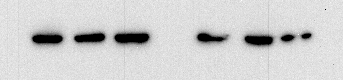
β-actin


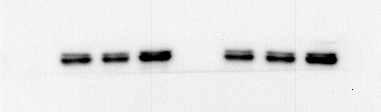


p-GSK3β

**(HepG2)**


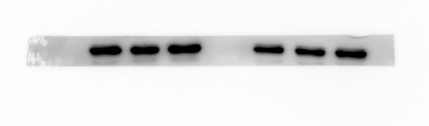


GSK3β


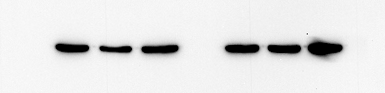


β-actin


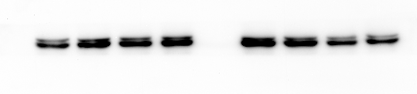
**B**

p-GSK3β


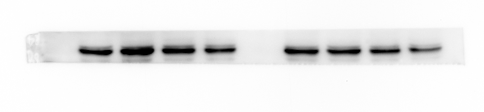


**(Huh-7)**

GSK3β


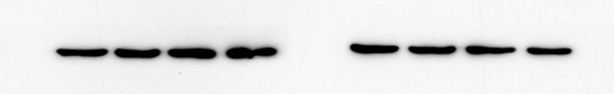


β-actin


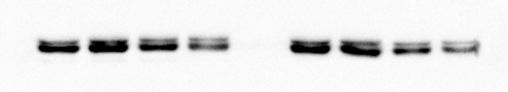


p-GSKβ


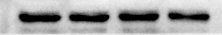


**(HepG2)**

GSK3β


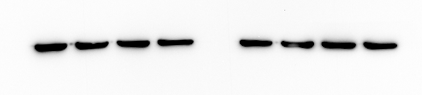


β-actin


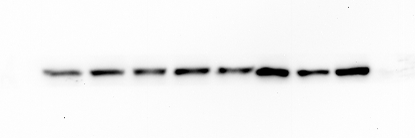
**F**

p-GSK3β


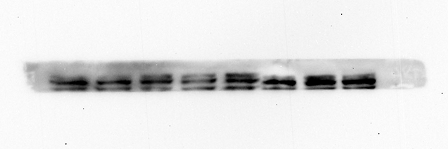


GSK3β

**
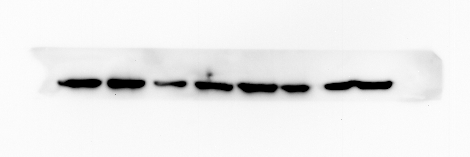
**

β-actin


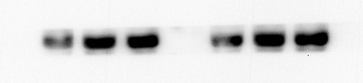
**H**

PD-L1

**(Huh-7)**


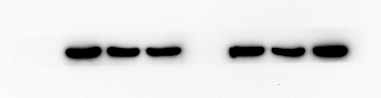
β-actin


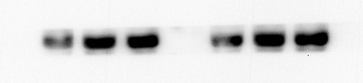
PD-L1

**(HepG2)**


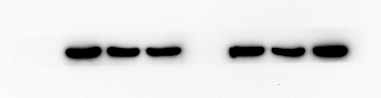


β-actin

**Figure 6**


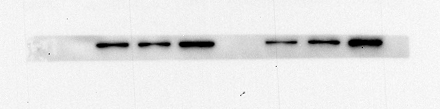
**A**

β-catenin

**(Huh-7)**


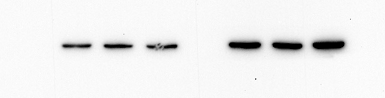


β-actin


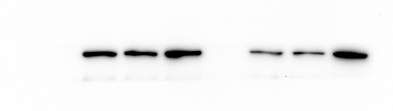


β-catenin

**(HepG2)**


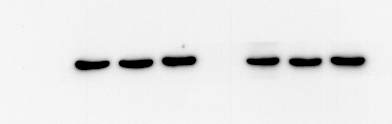


β-actin


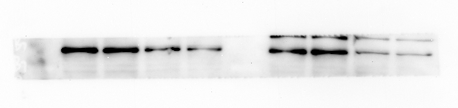
**B**

β-catenin


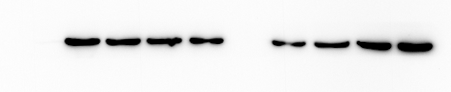


**(Huh-7)**

β-actin


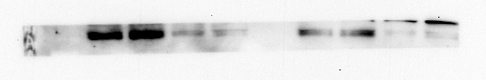


β-catenin


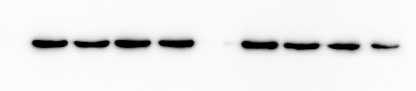


**(HepG2)**

β-actin


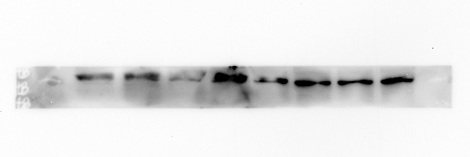


**F**

β-catenin


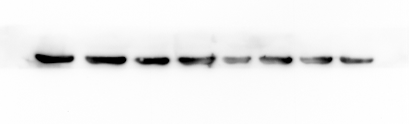


β-actin


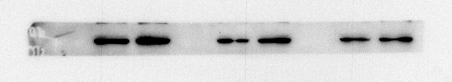
**G**

β-catenin


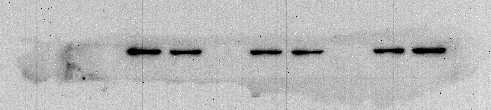


**(Huh-7)**

Lamin B1


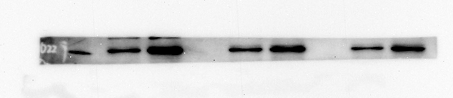


β-catenin

**(HepG2)**


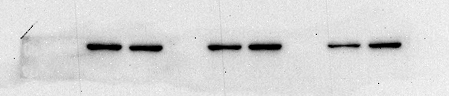


Lamin B1


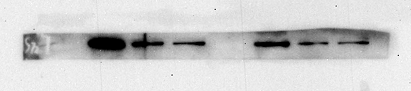
**H**

β-catenin

**(Huh-7)**

**
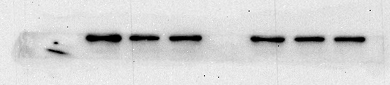
**

Lamin B1


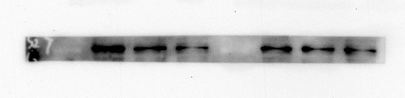


β-catenin

**(HepG2)**


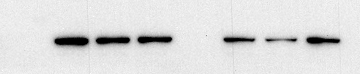


Lamin B1

**Figure 7**


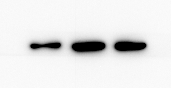
**A**

β-catenin

**(Huh-7)**


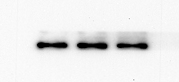


β-actin


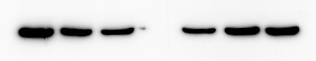


β-catenin

**(HepG2)**


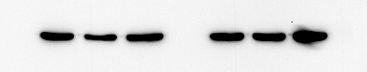


β-actin


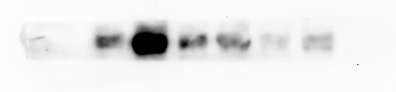
**C**

PD-L1

**(Huh-7)**


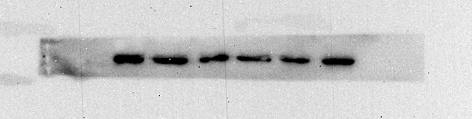


β-actin


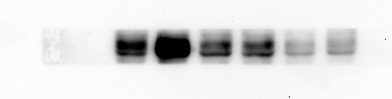


PD-L1

**(HepG2)**


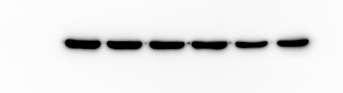


β-actin


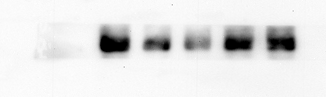
**E**

PD-L1

**(Huh-7)**


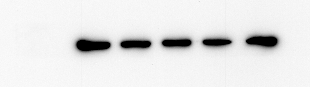


β-actin


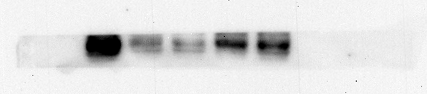


PD-L1

**(HepG2)**


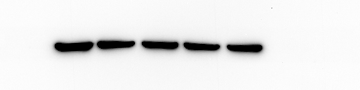


β-actin
